# Changes in Thyroid Function and Autoimmunity in Older Individuals: Longitudinal Analysis of the Whickham Cohort

**DOI:** 10.1210/clinem/dgae875

**Published:** 2024-12-14

**Authors:** Salman S Razvi, Helen Wild, Lorna Ingoe, Jonathan Vernazza, Mark Vanderpump, Simon H S Pearce, Marian Ludgate

**Affiliations:** Translational and Clinical Research Institute, Newcastle University Centre for Life, Central Parkway, Newcastle upon Tyne NE1 3BZ, UK; Department of Endocrinology, Gateshead Health NHS Foundation Trust, Gateshead NE9 6SX, UK; Department of Endocrinology, Gateshead Health NHS Foundation Trust, Gateshead NE9 6SX, UK; Department of Endocrinology, Gateshead Health NHS Foundation Trust, Gateshead NE9 6SX, UK; Department of Endocrinology, Gateshead Health NHS Foundation Trust, Gateshead NE9 6SX, UK; OneWelbeck Endocrinology, 1 Wellbeck Street, London W1G 0AR, UK; Translational and Clinical Research Institute, Newcastle University Centre for Life, Central Parkway, Newcastle upon Tyne NE1 3BZ, UK; Thyroid Research Group, Division of Infection and Immunity, Cardiff University School of Medicine, Cardiff CF14 4XN, UK

**Keywords:** thyroid function, aging, TSH

## Abstract

**Background:**

Longitudinal studies of thyroid function have demonstrated differing results. It remains unclear whether changes in thyroid function affect the diagnosis of subclinical thyroid dysfunction with aging.

**Methods:**

Survivors of the Whickham cohort study were evaluated on 2 occasions between the years 2008 and 2012 and 2016 and 2019. Serum TSH, free T4 (FT4), free T3 (FT3), and thyroid peroxidase antibody (TPOAb) were measured on both occasions using the same assay under similar conditions. Individuals with known thyroid disease or on medications affecting thyroid function were excluded. Comorbidities were noted, functional mobility was assessed by the timed up-and-go test, and muscle function was evaluated by the hand grip strength test.

**Results:**

In 204 individuals (mean age 77.0 [±6.6] years, 114 [56%] female), followed over a median (interquartile range) of 7.8 (7.3-8.2) years, serum TSH increased by 0.29 mU/L (12.4%), FT3 and TPOAb reduced by 0.1 pmol/L (−2.1%) and 0.6 U/L (−11.2%), and there were no significant changes in FT4 levels. The calculated upper limit of serum TSH increased over the follow-up period from 4.74 mU/L to 6.28 mU/L. The relationship between serum TSH and FT4 at both time points was not significantly different. Utilizing standard laboratory reference ranges, the prevalence of subclinical hypothyroidism increased from 3.5% at baseline to 9.0% at follow-up. However, adopting a visit-specific TSH reference range reduced the prevalence of subclinical hypothyroidism at both time points to 2.0%.

**Discussion:**

Thyroid function demonstrates subtle but significant changes with age. Utilizing standard reference ranges tends to increase the diagnosis of subclinical hypothyroidism in older euthyroid individuals. Our data suggest that adopting age-appropriate TSH reference ranges may reduce the risk of diagnosing and (potentially unnecessarily) treating subclinical hypothyroidism.

Thyroid function changes with age (1). Several studies have shown that older individuals have higher serum TSH levels than their younger counterparts (2-5), although the mechanisms underlying these changes are unclear. Furthermore, slightly higher serum TSH levels in older individuals are not associated with adverse clinical outcomes or reduced longevity (6-8).

Longitudinal cohort studies that have assessed variations in thyroid function over time have shown conflicting results. Some studies have demonstrated a rise in serum TSH and/or free T4 (FT4) levels over time, while others have revealed no changes or reductions in TSH or FT4 (9-13). The differences observed in various studies could be due to several reasons including a lack of assessment of thyroid autoimmunity, not assessing the impact of concomitant diseases and medications, a relatively short follow-up, the iodine status of the population being studied, or a survivor effect as those with lower TSH levels may have higher mortality. The change observed in serum TSH and FT4 with age can impact their respective reference ranges (14). The current practice of utilizing a standard reference range across all adult age groups may lead to a disproportionately higher proportion of older individuals being diagnosed with, and potentially treated for, subclinical hypothyroidism (15). Treatment of potentially age-related physiological TSH elevation could be one of the reasons why trials of levothyroxine in older individuals with mild subclinical hypothyroidism have shown no benefit in improving quality of life or symptoms (16, 17).

We, therefore, analyzed longitudinal clinical and biochemical data from the survivors of the Whickham cohort—the longest-running longitudinal cohort study related to thyroid function—to assess changes in serum thyroid function parameters and thyroid autoimmunity and its relationship with health and disease.

## Materials and Methods

### Participants

The Whickham cohort, a community-based population originally surveyed between 1972 and 1974, has been described previously (18-20). For this analysis, survivors of the original cohort were invited to attend a study visit between 2008 and 2012 and a follow-up visit between 2016 and 2019. Individuals with known thyroid disease, being treated with medications that affect thyroid function, or with newly diagnosed overt thyroid dysfunction [defined as abnormal serum TSH and FT4 or free T3 (FT3)] were excluded. At both study visits, participants attended in the morning after an overnight fast, and detailed physical, social, and medical assessments were undertaken. At the follow-up visit, additional assessments of mobility and muscle strength were performed. Mobility was evaluated by the timed up-and-go (TUG) test (21) and muscle strength was estimated by the hand grip strength (HGS) with a dynamometer (22). The burden of comorbid medical conditions was noted by calculating the Charlson Comorbidity Index (CCI) (23). Participants who could not attend the study center were assessed in their homes to reduce healthy user bias. Written informed consent was obtained from each participant, and the study was approved by the Newcastle and North Tyneside Research Ethics Committee.

### Biochemical Analysis

Serum TSH, FT4, FT3, and thyroid peroxidase antibody (TPOAb) at both visits were evaluated by the same method using the Roche Elecsys electrochemiluminescence immunoassay on the Cobas e602 platform. TSH receptor antibody (TRAb) was measured (Roche e-cobas E7100) only if serum TSH level was < 1.0 mU/L. The laboratory reference ranges for each of the analytes were: TSH (0.3-4.5 mU/L), FT4 (10-22 pmol/L), and FT3 (3.5-6.5 pmol/L). TPOAb and TRAb were classed as positive if >35 U/L and 1.8 U/L, respectively.

### Statistical Analysis

Variables that were not uniformly distributed are reported as median (interquartile range (IQR) and those that were uniformly distributed as mean (±SD). The paired *t*-test was used to assess differences in thyroid function and TPOAb parameters. Parameters that were not uniformly distributed were log-transformed before analyses. Multiple linear regression analysis was performed to assess the relationship between relevant variables and changes in thyroid function and TPOAb levels. In the primary analysis, changes in thyroid function (delta TSH, delta FT4, and delta FT3) were the dependent variables, whereas baseline age, sex, smoking status, body mass index (BMI), CCI, and baseline TPOAb levels (and delta FT4 or delta FT3 for the analysis of delta TSH) were the independent variables used in the regression model. In secondary analyses, TUG or HGS were the dependent variables and baseline age; sex; BMI; smoking status; and changes in TSH, FT4, or FT3 were the independent variables. Collinearity was assessed by variance inflation factor and the normal distribution of residuals by a visual inspection of the P-P plot of regression standardized residuals. The relationship between serum TSH and FT4 at both time points was evaluated by comparing the linear regression slopes of a scatterplot with TSH on the x-axis and FT4 on the y-axis. The statistical package SPSS v21.0 (Chi, Ill) was used to perform all statistical analyses.

## Results

In the original survey between 1972 and 1974, 2779 individuals were evaluated (mean age of 47 years; 54% female). At the 20-year follow-up, 1704 survivors were tested whose mean age was 58 years, of whom 56% were female. Since then, a further 814 people were deceased and 686 individuals had either moved away, were not capable of providing informed consent, or did not respond to the study invitation letter. Thus, a total of 204 cohort survivors participated in this study. The mean (±SD) age of the participants was 77.0 (±6.6) years, 114 (55.9%) were female, the mean BMI was 27.8 (±5.1) kg/m^2^, and 5 individuals (2.5%) were current smokers. Participants were assessed at follow-up after a median (IQR) interval of 7.8 (7.3-8.2) years.

### Change in TFTs and TPOAb Over Time

**Figure 1.**
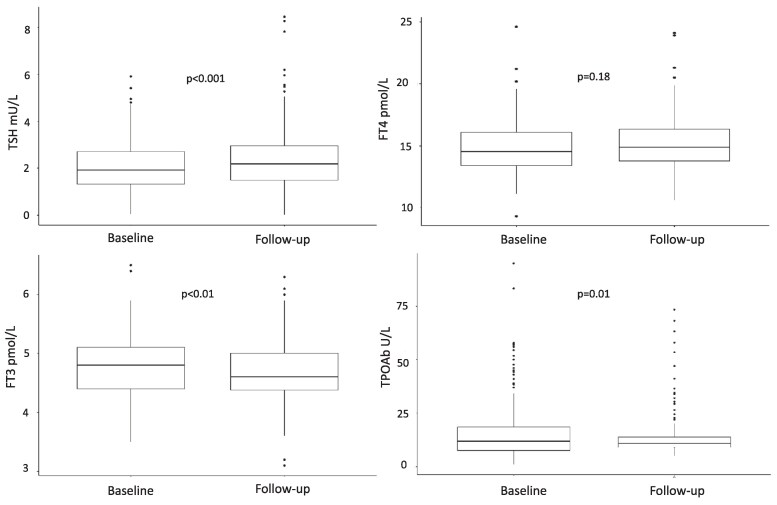
Longitudinal change in thyroid function and TPOAb over time. The line in the box represents the median value; the lower edge of the box represents the lower quartile and the upper edge of the box shows the upper quartile. The values at which the horizontal lines stop are the values of the upper and lower values of the data. The single points on the diagram show the outliers. The *P*-values were obtained from paired *t*-test analyses (TSH and TPOAb were log-transformed prior to analysis). Abbreviations: TPOAb, thyroid peroxidase antibody.

Between baseline and the follow-up visits, median (IQR) TSH increased significantly from 1.94 mU/L (1.35-2.73) to 2.18 mU/L (1.50-3.01), *P* < .001 **(**Fig. 1**)** (+12.4%). Mean (±SD) FT4 levels remained unchanged from 14.9 pmol/L (±2.1) to 15.1 pmol/L (±2.1), *P* = .18, whereas mean (±SD) FT3 levels reduced from 4.77 pmol/L (±0.54) to 4.67 pmol/L (±0.53), *P* < .01 (Fig. 1), (−2.1%). Median (IQR) TPOAb levels reduced significantly over time from 12.5 U/L (7.6-21.3) to 11.1 U/L (9.0-14.7), *P* = .01 (−11.2%). Thirty-one individuals (15.2%) had positive TPOAb levels at baseline and only 18 (8.8%) remained in a TPOAb-positive state.

**Figure 2.**
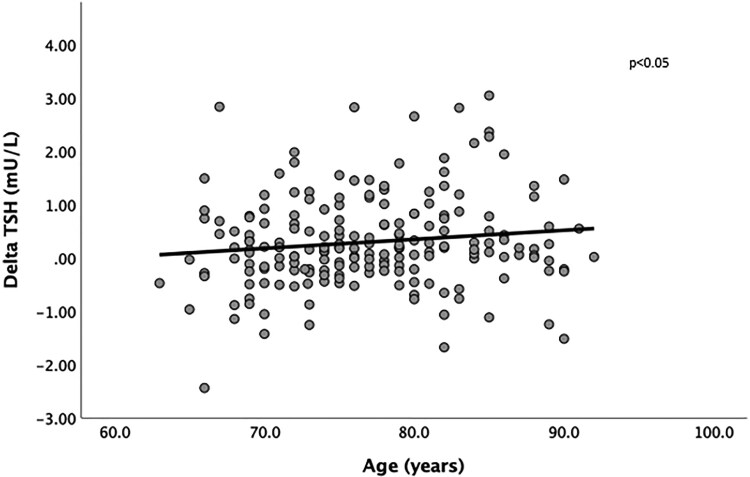
The relationship between longitudinal change in TSH with baseline age. Scatterplot with fitted regression line. The *P-*value was derived from a multivariable linear regression analysis that included baseline age, sex, smoking status, body mass index, Charlson Comorbidity Index, baseline thyroid peroxidase antibody levels, and delta free FT4 or delta free T3.

Changes in serum TSH were positively associated with baseline age: each year associated with a 0.02 (0.001-0.05) mU/L increase over time (Fig. 2). For baseline TPOAb levels, each 1 U/L increase was associated with a 0.001 (0.000-0.002) mU/L increase, and in a negative relationship with CCI, each unit increase in score was associated with a −0.07 (−0.16−0.00) mU/L reduction. In addition, delta TSH was also independently positively associated with delta FT3: for each 1 pmol/L increase in serum FT3 over time, serum TSH increased by 0.31 (0.08-0.55) mU/L. However, the significant association between baseline TPOAb levels and delta TSH was no longer present when 2 individuals with markedly high TPOAb levels (>500 U/L) were excluded from this analysis. There was also no significant relationship between baseline serum TSH and delta TSH. There were no significant predictors of changes in serum FT4, FT3, or TPOAb over time.

**Figure 3.**
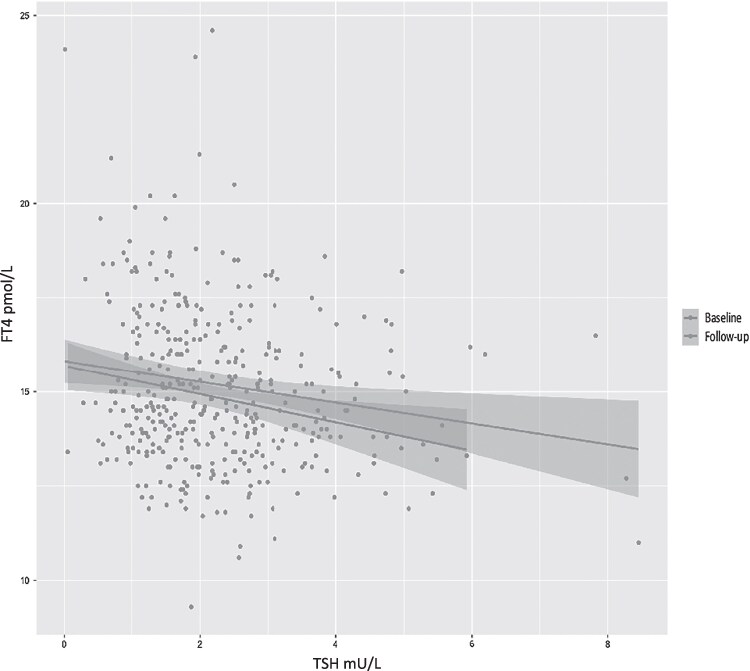
The relationship between serum TSH and free T4 levels at each visit. The slopes of the regression line assessing the relationship between TSH and free T4 at both time points were not statistically different.

As expected, there was a negative relationship between serum TSH and FT4 at both time points (r = −0.20 and −0.18, at baseline and follow-up, respectively), which did not change significantly over the follow-up period (Fig. 3). There was no significant relationship between changes in thyroid hormones (neither FT4 nor FT3) with changes in serum TSH over time.

### Changes in TFT Reference Ranges and Impact on the Diagnosis of Thyroid Dysfunction

**Figure 4.**
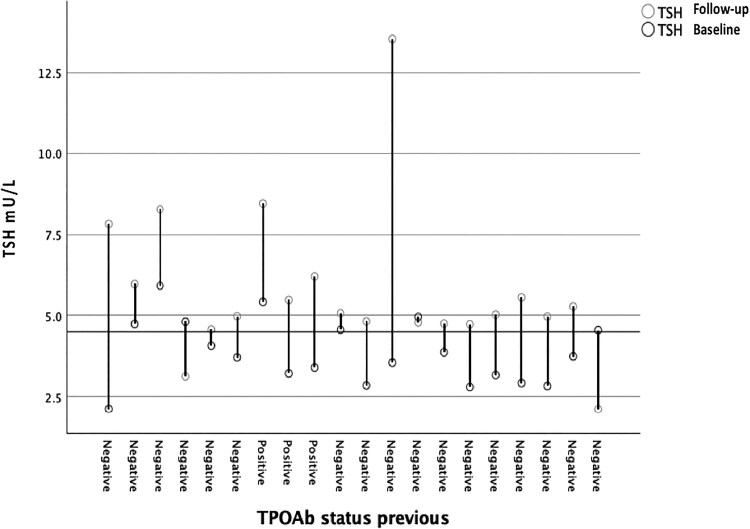
Change in serum TSH levels in individuals with subclinical hypothyroidism and their baseline thyroid peroxidase antibody status.

None of the participants had overt thyroid dysfunction at baseline or the follow-up visit. The prevalence of subclinical hypothyroidism, computed using standard TSH reference ranges, increased from 3.5% at baseline to 9.0% at follow-up (Table 1). Of the 7 individuals with subclinical hypothyroidism at baseline, 5 remained in the same state (of which only 1 was TPOAb positive) and 2 normalized serum TSH levels (both TPOAb negative) (Fig. 4) at follow-up. During the study period, 13 individuals with normal thyroid function at baseline, of which 12 were TPOAb negative, developed subclinical hypothyroidism (Fig. 4). Subclinical hyperthyroidism, on the other hand, remained unchanged at 1% at both visits. Of the 2 individuals with subclinical hyperthyroidism at baseline, 1 normalized serum TSH (TPOAb and TRAb negative) and 1 remained in the same state (TPOAb and TRAb negative). One person who was euthyroid at baseline went on to become subclinical hyperthyroid at the follow-up visit (TPOAb positive and TRAb negative).

**Table 1.** Prevalence of subclinical thyroid dysfunction at baseline and follow-up visits calculated using standard or visit-specific TSH reference range

	Standard TSH reference range (0.3-4.5 mU/L)		Visit-specific TSH reference range	
	Baseline	Follow-up	Baseline (0.64-4.74)	Follow-up (0.54-6.28)
Euthyroidism, n (%)	191 (95.5)	180 (90.0)	192 (96.0)	192 (96.0)
Subclinical hypothyroidism, n (%)	7 (3.5)	18 (9.0)	4 (2.0)	4 (2.0)
Subclinical hyperthyroidism, n (%)	2 (1.0)	2 (1.0)	4 (2.0)	4 (2.0)

The standard TSH reference range is laboratory-provided and the visit-specific TSH reference range is calculated as the 2.5th-97.5th percentile.

However, when visit-specific reference ranges were applied, the upper limit of TSH increased from 4.74mU/L at baseline to 6.28mU/L at follow-up (Table 1). Consequently, the prevalence of subclinical hypothyroidism remained unchanged at 2.0% at both visits. The lower limit of TSH also changed when visit-specific reference ranges were utilized: from 0.64mU/L to 0.54mU/L. The prevalence of subclinical hyperthyroidism was also unchanged at 2.0% at both visits.

### Association of Changes in TFT With Health and Disease

There was no relationship between TUG and delta TSH or delta FT3, but a positive relationship between TUG and delta FT4 was detected (for each 1 pmol/L rise in serum FT4, TUG increased by 0.04 seconds [0.01-0.07], *P* < .05). There was no association between HGS and delta TSH, FT4, or FT3 levels.

## Discussion

This prospective longitudinal study of older participants of the Whickham cohort has demonstrated small but clinically significant changes in thyroid function parameters over time. Serum TSH levels rose by 12.4% and FT3 concentrations reduced by 2.1% over 8 years in healthy euthyroid individuals. Furthermore, aging-related increases impact the upper limit of the serum TSH reference range, and this has implications for the diagnosis of subclinical hypothyroidism.

Several longitudinal studies have assessed changes in thyroid function over time, and their results have been conflicting. The Busselton Health survey, a longitudinal study of 1100 middle-aged participants (mean age 46 years) followed over 13 years, showed a 21.5% increase in TSH with ageing with no change in FT4 levels (9). Similar to our data, the most marked increase in TSH was observed in older individuals. The Cardiovascular Health All-Stars study in 843 older participants (average age of 85 years) demonstrated a 13% rise in TSH, a 1.7% increase in FT4, and a 13% reduction in total T3 over 13 years of follow-up (10). Furthermore, the minor elevation in TSH was not associated with mortality although higher FT4 was linked with death. The Tromso study also showed a rise in TSH with age; nonsmokers revealed a bigger increase than smokers (24). In the Tehran thyroid study of 1755 middle-aged (mean age 44 years) individuals, TSH levels increased whereas FT4 concentrations reduced over 10 years (11).

Contrary to these results, the Rotterdam study in 1225 older participants (average age 67 years) followed over 6.5 years showed no change in TSH whereas FT4 levels increased over time (25). Similarly, in the Nijmegen Biomedical study of older individuals (mean age 61 years), serum TSH was reduced by 5.4% and FT4 increased by 3.7% over 4 years in 980 participants (12). It is not readily clear what may explain the disparity in results. One possible reason could be that parts of the Netherlands were known to be iodine-deficient in the past (26). In comparison, the UK population is deemed to be broadly iodine-sufficient, although some studies have suggested that school girls aged 14 to 15 years may be mildly deficient (27, 28).

This analysis has not elucidated the mechanisms underlying changes in thyroid function with age. We had originally planned to evaluate serum TSH bioactivity using luciferase bioassays (29), but the quantity of serum available was insufficient to obtain meaningful results. Nevertheless, our data provides some clues, and several possible explanations are conceivable. One possibility is that the pituitary produces more biologically inactive TSH with age. Thus, immunoassays may detect a higher circulating TSH level but the stimulatory effect on the thyroid remains unchanged as evidenced by unchanged FT4 levels. The other possible explanation is that either the thyroid gland or the pituitary/hypothalamic complex becomes more resistant to TSH or thyroid hormone levels, respectively, with age, leading to higher circulating TSH (30). Our results reveal that increases in serum TSH levels are positively related to higher circulating FT3 levels, probably because the intrathyroidal activities of both deiodinase 1 and 2 are stimulated by TSH (31). Thus, it is unlikely that the higher TSH levels are related to negative feedback alterations in circulating thyroid hormone concentrations. The minor reduction in TPOAb levels with age confirms that the rise in TSH is unrelated to the progression of autoimmune processes. The negative relationship between disease burden, as quantified by the CCI score, and serum TSH levels suggests that chronic nonthyroidal illness may dampen some of the rise in TSH with age in older people.

This analysis did not reveal any significant associations of changes in serum TSH or FT3 with mobility or muscle strength. Higher FT4 levels, however, were associated with worse mobility, which is consistent with findings from another cohort that assessed gait and mobility in older individuals (32).

This analysis has several strengths. All participants were studied identically several years apart, with the same assay, in a fasting state and at similar times. In addition, individuals with known thyroid disease or those on medications affecting thyroid function were excluded. Thyroid autoimmunity was evaluated at both periods and occult Graves’ disease was assessed (and excluded) in individuals with low or low-normal TSH levels. Furthermore, this is the first analysis, to our knowledge, that has assessed the determinants of changes in thyroid function including comorbidities and the relationship between changes in thyroid function parameters with mobility and muscle strength. Our study also has limitations. A relatively small number of participants were studied compared to some other cohorts. In addition, we did not examine the mechanisms that explain the observed changes in thyroid function parameters. Similar to other longitudinal studies, our analysis also suffers from a survivor selection bias as the sample consisted of individuals identified from the previous Whickham survey. It is, therefore, possible that individuals with a higher risk of morbidity and mortality or thyroid dysfunction at baseline were excluded, which limits the generalisability of our findings to the wider population with multiple comorbidities. For example, individuals with lower serum TSH levels at baseline due to chronic nonthyroidal illness are more likely to be absent at the follow-up visit, thus potentially contributing to the observed rise in serum TSH over time. However, we have considered this aspect by adjusting for comorbidities in our analyses.

In conclusion, our study has demonstrated that thyroid function changes over time. These results confirm that the upper limit of serum TSH rises with age. If age-specific TSH reference ranges are not used, then a large number of otherwise euthyroid individuals would be diagnosed with subclinical hypothyroidism, and some potentially could be commenced on unnecessary treatment.

## Data Availability

Some or all datasets generated during and/or analyzed during the current study are not publicly available but are available from the corresponding author on reasonable request.
